# Diethyl 2-[(4-bromo­anilino)methyl­idene]malonate

**DOI:** 10.1107/S1600536810045150

**Published:** 2010-11-10

**Authors:** Zhi-Qiang Feng, Xiao-Li Yang, Yuan-Feng Ye, Tao Dong, Huai-Qing Wang

**Affiliations:** a College of Materials Engineering, Jinling Institute of Technology, No. 99 Hongjing Street, Nanjing 211169, People’s Republic of China

## Abstract

In the title compound, C_14_H_16_BrNO_4_, inter­molecular C—H⋯O hydrogen bonds link the mol­ecules, forming a stable structure. An intra­molecular N—H⋯O hydrogen bond results in the formation of a six-membered ring and helps to establish the mol­ecular conformation which is almost planar, with an r.m.s deviation of 0.0842 Å.

## Related literature

For the preparation, see: Lager *et al.* (2006 ▶). For bond-length data, see: Allen *et al.* (1987 ▶).
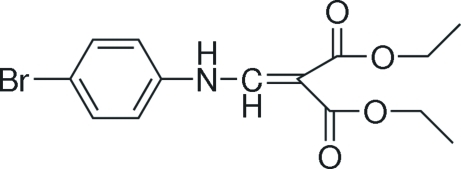


## Experimental

### 

#### Crystal data

C_14_H_16_BrNO_4_
*M* *_r_* = 342.19
Monoclinic, 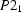

*a* = 9.2440 (18) Å
*b* = 6.5000 (13) Å
*c* = 13.448 (3) Å
β = 110.10 (3)°
*V* = 758.8 (3) Å^3^
*Z* = 2
Mo *K*α radiation
μ = 2.72 mm^−1^
*T* = 293 K
0.30 × 0.10 × 0.10 mm

#### Data collection

Enraf–Nonius CAD-4 diffractometer
Absorption correction: ψ scan (North *et al.*, 1968 ▶) *T*_min_ = 0.496, *T*_max_ = 0.773
2851 measured reflections
2790 independent reflections
1606 reflections with *I* > 2σ(*I*)
*R*_int_ = 0.071
3 standard reflections every 200 reflections  intensity decay: 1%

#### Refinement

*R*[*F* ^2^ > 2σ(*F* ^2^)] = 0.066
*wR*(*F* ^2^) = 0.154
*S* = 1.00
2790 reflections
181 parameters
1 restraint
H-atom parameters constrained
Δρ_max_ = 0.49 e Å^−3^
Δρ_min_ = −0.39 e Å^−3^
Absolute structure: Flack (1983 ▶), 1253 Friedel pairs
Flack parameter: −0.01 (2)

### 

Data collection: *CAD-4 EXPRESS* (Enraf–Nonius, 1989 ▶); cell refinement: *CAD-4 EXPRESS*; data reduction: *XCAD4* (Harms & Wocadlo, 1995 ▶); program(s) used to solve structure: *SHELXS97* (Sheldrick, 2008 ▶); program(s) used to refine structure: *SHELXL97* (Sheldrick, 2008 ▶); molecular graphics: *SHELXL97*; software used to prepare material for publication: *PLATON* (Spek, 2009 ▶).

## Supplementary Material

Crystal structure: contains datablocks global, I. DOI: 10.1107/S1600536810045150/bq2249sup1.cif

Structure factors: contains datablocks I. DOI: 10.1107/S1600536810045150/bq2249Isup2.hkl

Additional supplementary materials:  crystallographic information; 3D view; checkCIF report

## Figures and Tables

**Table 1:** Hydrogen-bond geometry (Å, °)

*D*—H⋯*A*	*D*—H	H⋯*A*	*D*⋯*A*	*D*—H⋯*A*
N—H0*A*⋯O3	0.86	1.95	2.615 (8)	134
C1—H1*A*⋯O3^i^	0.93	2.49	3.190 (10)	132
C5—H5*A*⋯O1^ii^	0.93	2.42	3.298 (9)	157

Symmetry codes: (i) 
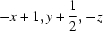
; (ii) 
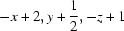
.

**Fig. 1.**
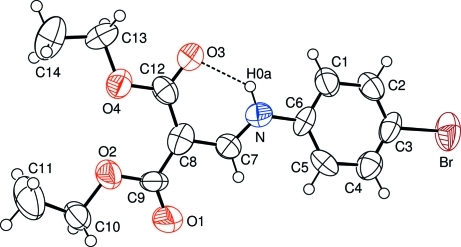
The molecular structure of the title molecule, with the atom-numbering scheme. Displacement ellipsoids are drawn at the 50% probability level. Dashed lines show H-bonding.

**Fig. 2.**
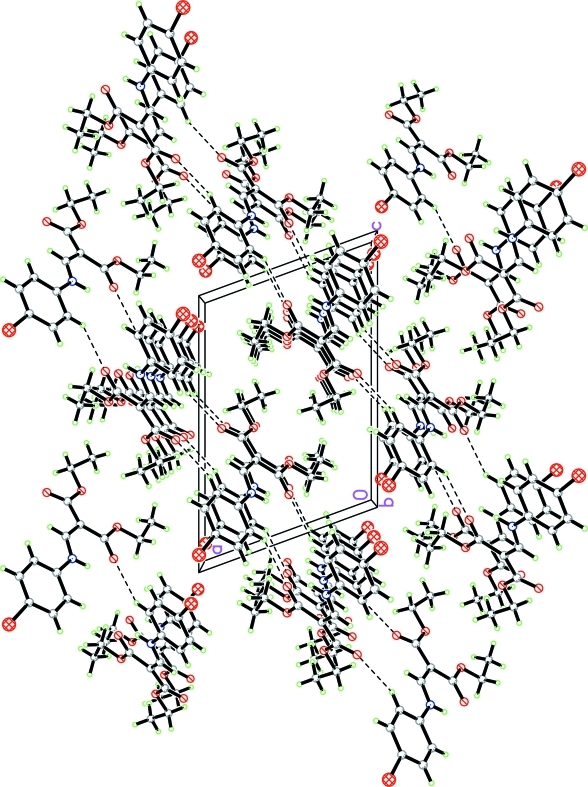
A packing diagram of the title compound. Hydrogen bonds are shown as dashed line.

## supplementary crystallographic information

### Comment

We report herein the crystal structure of the title compound, diethyl 2-((4-bromophenylamino)methylene)malonate which is an important intermediate of synthesizing pyrazoloquinolinones. In the molecule of the title compound (Fig. 1), all bond lengths and angles (Allen *et al.*, 1987) are within normal ranges. The intramolecular N-H···O hydrogen bond (Table 1) results in the formation of a six-membered ring (N/C7/C8/C12/O3/H0A). In the crystal structure, intermolecular weak C-H···O hydrogen bonds link the molecules to form a stable structure (Fig. 2).

### Experimental

The title compound, diethyl 2-((4-bromophenylamino)methylene)malonate was prepared by the literature method (Lager *et al.*, 2006). 4-bromoaniline (1.2 mmol) and diethyl ethoxymethylenemalonate (1.2 mmol) were mixed and heated at 403 K for 2 h. Low boiling components were evaporated at low pressure with a cold trap yielding diethyl 2-((4-bromophenylamino) methylene)malonate. The crude product was purified by recrystallization from diethyl ether yielding the title compound (73 % yield), as a white solid. Crystals suitable for X-ray analysis were obtained by slow evaporation of an methanol solution.

### Refinement

H atoms were positioned geometrically, with N-H = 0.86 Å (for NH) and C-H = 0.93, 0.98 and 0.96 Å for aromatic, methine and methyl H, respectively, and constrained to ride on their parent atoms, with U_iso_(H) = xU_eq_(C, N), where x = 1.5 for methyl H and x = 1.2 for all other H atoms.

### Crystal data

**Table:**

C_14_H_16_BrNO_4_	*F*(000) = 348
*M**_r_* = 342.19	*D*_x_ = 1.498 Mg m^−^^3^
Monoclinic, *P*2_1_	Melting point: 367 K
Hall symbol: P 2yb	Mo *K*α radiation, λ = 0.71073 Å
*a* = 9.2440 (18) Å	Cell parameters from 25 reflections
*b* = 6.5000 (13) Å	θ = 9–14°
*c* = 13.448 (3) Å	µ = 2.72 mm^−^^1^
β = 110.10 (3)°	*T* = 293 K
*V* = 758.8 (3) Å^3^	Needle, colourless
*Z* = 2	0.30 × 0.10 × 0.10 mm

### Data collection

**Table:**

Enraf–Nonius CAD-4 diffractometer	1606 reflections with *I* > 2σ(*I*)
Radiation source: fine-focus sealed tube	*R*_int_ = 0.071
graphite	θ_max_ = 25.4°, θ_min_ = 1.6°
ω/2θ scans	*h* = −11→11
Absorption correction: ψ scan (North *et al.*, 1968)	*k* = −7→7
*T*_min_ = 0.496, *T*_max_ = 0.773	*l* = −5→16
2851 measured reflections	3 standard reflections every 200 reflections
2790 independent reflections	intensity decay: 1%

### Refinement

**Table:**

Refinement on *F*^2^	Secondary atom site location: difference Fourier map
Least-squares matrix: full	Hydrogen site location: inferred from neighbouring sites
*R*[*F*^2^ > 2σ(*F*^2^)] = 0.066	H-atom parameters constrained
*wR*(*F*^2^) = 0.154	*w* = 1/[σ^2^(*F*_o_^2^) + (0.078*P*)^2^ + ] where *P* = (*F*_o_^2^ + 2*F*_c_^2^)/3
*S* = 1.00	(Δ/σ)_max_ < 0.001
2790 reflections	Δρ_max_ = 0.49 e Å^−^^3^
181 parameters	Δρ_min_ = −0.39 e Å^−^^3^
1 restraint	Absolute structure: Flack (1983), 1253 Friedel pairs
Primary atom site location: structure-invariant direct methods	Flack parameter: −0.01 (2)

### Special details

**Table:**

Geometry. All esds (except the esd in the dihedral angle between two l.s. planes) are estimated using the full covariance matrix. The cell esds are taken into account individually in the estimation of esds in distances, angles and torsion angles; correlations between esds in cell parameters are only used when they are defined by crystal symmetry. An approximate (isotropic) treatment of cell esds is used for estimating esds involving l.s. planes.
Refinement. Refinement of F^2^ against ALL reflections. The weighted R-factor wR and goodness of fit S are based on F^2^, conventional R-factors R are based on F, with F set to zero for negative F^2^. The threshold expression of F^2^ > 2sigma(F^2^) is used only for calculating R-factors(gt) etc. and is not relevant to the choice of reflections for refinement. R-factors based on F^2^ are statistically about twice as large as those based on F, and R- factors based on ALL data will be even larger.

### Fractional atomic coordinates and isotropic or equivalent isotropic displacement parameters (Å^2^)

**Table:**

	*x*	*y*	*z*	*U*_iso_*/*U*_eq_	
Br	0.96900 (11)	0.78434 (19)	0.08931 (9)	0.1008 (4)	
N	0.7057 (7)	0.0195 (9)	0.2183 (5)	0.0626 (16)	
H0A	0.6242	−0.0249	0.1697	0.075*	
C1	0.7111 (8)	0.2666 (15)	0.0881 (6)	0.0691 (19)	
H1A	0.6318	0.1944	0.0387	0.083*	
O1	0.8800 (8)	−0.2964 (10)	0.4901 (5)	0.116 (3)	
O2	0.7014 (6)	−0.5254 (9)	0.4487 (4)	0.0768 (15)	
C2	0.7701 (9)	0.4458 (13)	0.0594 (6)	0.068 (2)	
H2A	0.7280	0.4973	−0.0091	0.082*	
C3	0.8892 (9)	0.5442 (11)	0.1320 (8)	0.069 (2)	
O3	0.4955 (6)	−0.2676 (8)	0.1562 (5)	0.0859 (18)	
C4	0.9487 (9)	0.4794 (12)	0.2343 (7)	0.068 (2)	
H4A	1.0276	0.5532	0.2832	0.082*	
O4	0.4944 (6)	−0.5243 (8)	0.2634 (4)	0.0673 (13)	
C5	0.8899 (8)	0.2992 (19)	0.2660 (5)	0.066 (2)	
H5A	0.9301	0.2520	0.3354	0.079*	
C6	0.7740 (8)	0.1980 (10)	0.1930 (6)	0.0558 (19)	
C7	0.7510 (8)	−0.0856 (11)	0.3061 (6)	0.0521 (17)	
H7A	0.8348	−0.0334	0.3608	0.062*	
C8	0.6869 (8)	−0.2699 (11)	0.3269 (6)	0.062 (2)	
C9	0.7662 (9)	−0.3565 (13)	0.4292 (6)	0.0594 (19)	
C10	0.7736 (9)	−0.6258 (12)	0.5497 (6)	0.072 (2)	
H10A	0.7952	−0.5273	0.6071	0.086*	
H10B	0.8694	−0.6907	0.5524	0.086*	
C11	0.6597 (12)	−0.7843 (17)	0.5578 (9)	0.124 (5)	
H11A	0.7005	−0.8544	0.6244	0.186*	
H11B	0.6411	−0.8817	0.5011	0.186*	
H11C	0.5648	−0.7178	0.5530	0.186*	
C12	0.5544 (9)	−0.3509 (12)	0.2415 (6)	0.0586 (18)	
C13	0.3642 (9)	−0.6084 (13)	0.1829 (6)	0.079 (2)	
H13A	0.3922	−0.6543	0.1233	0.094*	
H13B	0.2832	−0.5063	0.1581	0.094*	
C14	0.3121 (12)	−0.7836 (14)	0.2315 (8)	0.105 (4)	
H14A	0.2224	−0.8442	0.1805	0.157*	
H14B	0.2872	−0.7363	0.2913	0.157*	
H14C	0.3927	−0.8844	0.2543	0.157*	

### Atomic displacement parameters (Å^2^)

**Table:**

	*U*^11^	*U*^22^	*U*^33^	*U*^12^	*U*^13^	*U*^23^
Br	0.0966 (6)	0.0669 (5)	0.1532 (9)	−0.0125 (6)	0.0612 (6)	0.0184 (7)
N	0.051 (4)	0.065 (4)	0.065 (4)	−0.003 (3)	0.011 (3)	0.000 (3)
C1	0.072 (5)	0.056 (5)	0.079 (5)	0.008 (5)	0.024 (4)	0.012 (5)
O1	0.098 (5)	0.119 (6)	0.091 (4)	−0.047 (4)	−0.019 (4)	0.038 (4)
O2	0.056 (3)	0.080 (4)	0.085 (4)	−0.008 (3)	0.013 (3)	0.031 (3)
C2	0.060 (5)	0.073 (5)	0.074 (5)	0.008 (4)	0.026 (4)	0.026 (4)
C3	0.057 (5)	0.045 (4)	0.108 (7)	−0.014 (4)	0.034 (5)	−0.009 (5)
O3	0.078 (4)	0.071 (5)	0.088 (4)	−0.021 (3)	0.002 (3)	0.013 (3)
C4	0.058 (5)	0.060 (5)	0.087 (6)	−0.003 (4)	0.026 (5)	−0.006 (5)
O4	0.067 (3)	0.059 (3)	0.077 (3)	−0.010 (3)	0.026 (3)	0.000 (3)
C5	0.064 (4)	0.072 (5)	0.058 (4)	0.022 (6)	0.014 (4)	0.023 (5)
C6	0.059 (4)	0.042 (3)	0.076 (5)	−0.016 (3)	0.036 (4)	−0.015 (4)
C7	0.047 (4)	0.055 (4)	0.055 (5)	0.007 (3)	0.019 (4)	0.002 (3)
C8	0.052 (4)	0.058 (6)	0.088 (6)	−0.002 (3)	0.040 (4)	−0.008 (4)
C9	0.052 (5)	0.074 (5)	0.040 (4)	−0.009 (4)	0.001 (4)	0.000 (4)
C10	0.061 (5)	0.075 (5)	0.072 (5)	0.003 (4)	0.013 (4)	0.028 (4)
C11	0.087 (6)	0.132 (11)	0.157 (9)	0.002 (6)	0.048 (7)	0.075 (9)
C12	0.061 (5)	0.063 (5)	0.058 (5)	0.011 (4)	0.029 (4)	0.014 (4)
C13	0.065 (5)	0.086 (6)	0.073 (5)	−0.022 (5)	0.009 (4)	−0.022 (5)
C14	0.118 (8)	0.083 (7)	0.140 (9)	−0.035 (6)	0.080 (7)	−0.024 (6)

### Geometric parameters (Å, °)

**Table:**

Br—C3	1.898 (7)	C5—C6	1.349 (11)
N—C7	1.302 (8)	C5—H5A	0.9300
N—C6	1.417 (9)	C7—C8	1.407 (9)
N—H0A	0.8600	C7—H7A	0.9300
C1—C2	1.396 (11)	C8—C9	1.432 (10)
C1—C6	1.401 (10)	C8—C12	1.459 (11)
C1—H1A	0.9300	C10—C11	1.503 (11)
O1—C9	1.157 (8)	C10—H10A	0.9700
O2—C9	1.320 (9)	C10—H10B	0.9700
O2—C10	1.446 (8)	C11—H11A	0.9600
C2—C3	1.356 (11)	C11—H11B	0.9600
C2—H2A	0.9300	C11—H11C	0.9600
C3—C4	1.361 (11)	C13—C14	1.474 (11)
O3—C12	1.215 (8)	C13—H13A	0.9700
C4—C5	1.417 (14)	C13—H13B	0.9700
C4—H4A	0.9300	C14—H14A	0.9600
O4—C12	1.333 (8)	C14—H14B	0.9600
O4—C13	1.423 (8)	C14—H14C	0.9600
			
C7—N—C6	128.0 (6)	O1—C9—C8	126.0 (8)
C7—N—H0A	116.0	O2—C9—C8	113.6 (7)
C6—N—H0A	116.0	O2—C10—C11	105.6 (7)
C2—C1—C6	118.6 (8)	O2—C10—H10A	110.6
C2—C1—H1A	120.7	C11—C10—H10A	110.6
C6—C1—H1A	120.7	O2—C10—H10B	110.6
C9—O2—C10	117.9 (6)	C11—C10—H10B	110.6
C3—C2—C1	119.5 (8)	H10A—C10—H10B	108.8
C3—C2—H2A	120.3	C10—C11—H11A	109.5
C1—C2—H2A	120.3	C10—C11—H11B	109.5
C2—C3—C4	121.9 (7)	H11A—C11—H11B	109.5
C2—C3—Br	118.3 (7)	C10—C11—H11C	109.5
C4—C3—Br	119.8 (6)	H11A—C11—H11C	109.5
C3—C4—C5	119.8 (7)	H11B—C11—H11C	109.5
C3—C4—H4A	120.1	O3—C12—O4	120.0 (7)
C5—C4—H4A	120.1	O3—C12—C8	124.4 (7)
C12—O4—C13	117.7 (6)	O4—C12—C8	115.6 (6)
C6—C5—C4	118.3 (7)	O4—C13—C14	106.2 (7)
C6—C5—H5A	120.8	O4—C13—H13A	110.5
C4—C5—H5A	120.8	C14—C13—H13A	110.5
C5—C6—C1	121.9 (7)	O4—C13—H13B	110.5
C5—C6—N	122.1 (7)	C14—C13—H13B	110.5
C1—C6—N	116.0 (7)	H13A—C13—H13B	108.7
N—C7—C8	126.9 (7)	C13—C14—H14A	109.5
N—C7—H7A	116.5	C13—C14—H14B	109.5
C8—C7—H7A	116.5	H14A—C14—H14B	109.5
C7—C8—C9	114.5 (7)	C13—C14—H14C	109.5
C7—C8—C12	116.6 (7)	H14A—C14—H14C	109.5
C9—C8—C12	128.9 (6)	H14B—C14—H14C	109.5
O1—C9—O2	120.3 (7)		
			
C6—C1—C2—C3	2.2 (11)	C10—O2—C9—O1	2.6 (12)
C1—C2—C3—C4	−3.4 (11)	C10—O2—C9—C8	179.5 (6)
C1—C2—C3—Br	178.8 (6)	C7—C8—C9—O1	−5.5 (11)
C2—C3—C4—C5	2.7 (12)	C12—C8—C9—O1	171.8 (9)
Br—C3—C4—C5	−179.5 (6)	C7—C8—C9—O2	177.9 (6)
C3—C4—C5—C6	−0.8 (12)	C12—C8—C9—O2	−4.9 (11)
C4—C5—C6—C1	−0.3 (11)	C9—O2—C10—C11	169.0 (7)
C4—C5—C6—N	−178.4 (7)	C13—O4—C12—O3	1.8 (9)
C2—C1—C6—C5	−0.4 (11)	C13—O4—C12—C8	179.5 (6)
C2—C1—C6—N	177.8 (6)	C7—C8—C12—O3	−1.7 (10)
C7—N—C6—C5	−8.8 (11)	C9—C8—C12—O3	−178.9 (7)
C7—N—C6—C1	173.0 (7)	C7—C8—C12—O4	−179.3 (5)
C6—N—C7—C8	−175.6 (6)	C9—C8—C12—O4	3.5 (10)
N—C7—C8—C9	176.9 (7)	C12—O4—C13—C14	−173.9 (6)
N—C7—C8—C12	−0.7 (9)		

### Hydrogen-bond geometry (Å, °)

**Table:**

*D*—H···*A*	*D*—H	H···*A*	*D*···*A*	*D*—H···*A*
N—H0A···O3	0.86	1.95	2.615 (8)	134
C1—H1A···O3^i^	0.93	2.49	3.190 (10)	132
C5—H5A···O1^ii^	0.93	2.42	3.298 (9)	157

Symmetry codes: (i) −*x*+1, *y*+1/2, −*z*; (ii) −*x*+2, *y*+1/2, −*z*+1.
